# Doing Qualitative Workplace Relationship Research: Current Trends, New Directions, and Quality Indicators

**DOI:** 10.3390/bs16030394

**Published:** 2026-03-09

**Authors:** Jimmie Manning

**Affiliations:** Communication Studies Department, University of Nevada, Mail Stop 0229, 1664 North Virginia Street, Reno, NV 89523, USA; jimmiem@unr.edu

**Keywords:** qualitative research, workplace relationships, Qualitative Relationship Reporting Framework (QRRF-4), sociomateriality, research quality and rigor, organizational studies

## Abstract

Workplace relationship research has expanded rapidly across multiple disciplines, yet qualitative approaches to studying workplace relationships often remain constrained by single-participant designs, organization-centered analyses, and lingering post-positivist assumptions about rigor. Responding to these limitations, this article offers a methodological and conceptual primer for qualitative workplace relationship research grounded in interpersonal, organizational, constitutive, and reflexive traditions. Drawing on recent scholarship, the article first reviews contemporary conceptualizations of workplace relationships and highlights trends in qualitative research over the past decade. It then advances three generative directions for future inquiry: dyadic and multiadic research designs that capture relational co-construction; sociomaterial analyses that attend to objects, documents, bodies, and spaces as relationally consequential; and topic-centered approaches that theorize emergent workplace phenomena from participant-centered perspectives rather than theory-down deduction. The article further addresses questions of quality and rigor by engaging Tracy’s “big-tent” criteria and introducing the Qualitative Relationship Reporting Framework (QRRF-4), a relationship-centered reporting aid designed to support transparency and ethical accountability without reducing qualitative rigor to procedural compliance. Taken together, this article serves as both a practical resource and a theoretical invitation, supporting rigorous, reflexive qualitative research that illuminates the communicatively constituted, materially situated, and meaning-centered dynamics of contemporary workplace life.

## 1. Doing Qualitative Workplace Relationship Research: Current Trends, New Directions, and Quality Indicators

The study of workplace relationships—sometimes referred to as workplace communication studies—is a broad and evolving area of inquiry that spans multiple disciplinary traditions, including social and personal relationships, organizational communication, management communication, business and professional communication, corporate communication, and leadership communication. Across these traditions, scholars have examined communicative practices that enable coordination, productivity, and professional effectiveness, often with an eye toward pragmatic application and organizational outcomes. At the same time, research in this area has increasingly recognized that work is not only a site of task accomplishment but also a deeply relational space in which identities, meanings, and social bonds are constituted through communication. It is within this intersection—where professional roles, institutional structures, and personal relationships converge—that workplace relationship research has emerged as a vital and generative area of scholarship.

Workplace relationship research focuses on the communicative processes through which people experience, negotiate, and make sense of relationships that arise in and through work contexts. Importantly, the term *workplace* signals more than an organizational setting or formal role structure. As Chory and Horan (2023) argue, the distinction between *organizational* and *workplace* relationships is conceptually meaningful. Whereas organizational relationships are often defined by formal roles (e.g., supervisor–subordinate, coworker, mentor–protégé) and institutional hierarchies, workplace relationships emphasize the lived, personal, and relational dimensions of working together. In this view, “relationships” are primary, with the workplace serving as the context in which those relationships are formed, sustained, strained, and sometimes transformed beyond formal job descriptions or institutional expectations.

This orientation aligns closely with calls in the current Special Issue where this article is featured, a Special Issue inviting scholarship that attends to the complex professional and personal dimensions of contemporary work life. As J. M. Harden Fritz and Majocha (2024) emphasize, contemporary workplace communication is shaped by rapidly changing conditions including global crises such as the COVID-19 pandemic, the expansion of nontraditional and remote work arrangements, advances in communication technologies, and the growing presence of artificial intelligence in organizational contexts. These developments intersect with enduring concerns about socialization, mentoring, teamwork, inclusion and exclusion, civility and incivility, professional identity, and the shifting boundaries between work and nonwork life. Together, they underscore the need for research that can account for complexity, context, and meaning as they are experienced by people navigating workplace relationships in real time.

This article approaches qualitative workplace relationship research from a joint interpersonal, organizational, constitutive, and reflexive tradition. From an interpersonal perspective, workplace relationships are understood not as simple byproducts of organizational structures but as *communicatively accomplished relationships* that matter in their own right. From a constitutive perspective, communication is not treated as a variable that influences relationships after the fact; rather, relationships, identities, and workplace realities are understood as coming into being through organizational interaction. From a reflexive perspective, qualitative inquiry acknowledges that knowledge is situated, partial, and shaped by the positions of both participants and researchers, making reflexivity a strength rather than a liability in the research process.

Qualitative research is particularly well suited to advancing this understanding of workplace relationships. Interpretive qualitative approaches foreground meaning-making as an ongoing, relational, and contextually embedded process (Manning & Kunkel, 2014a). Rather than seeking universal laws or decontextualized variables, qualitative relationship research attends to how people construct, negotiate, and contest meanings in everyday interaction. Such approaches allow researchers to observe meaning-making in action, elevate marginalized or understudied voices, engage emotion and lived experience, and examine how relationships are constituted across multiple levels of interaction, culture, and material context. These commitments are especially important in workplace settings, where power, hierarchy, identity, and institutional norms shape not only what relationships look like, but also how they are talked about, evaluated, and sanctioned.

Qualitative studies of workplace relationships also carry distinctive value within the broader landscape of organizational and interpersonal scholarship. As J. Harden Fritz (2014) demonstrates, the qualitative turn in organizational communication created generative ground for examining workplace relationships as socially constructed, contextually situated, and communicatively constituted phenomena. Qualitative workplace relationship research has illuminated the microprocesses of relating at work, revealed the emotional and ethical dimensions of professional interaction, and generated meaning-centered theories that would be difficult to access through post-positivist approaches alone. At the same time, Harden Fritz underscores that qualitative inquiry in this area remains unevenly distributed, often constrained by methodological expectations that privilege variable-based, outcome-oriented models of work relationships. Attending carefully to qualitative values, therefore, is not only a methodological choice but also an epistemological and ethical commitment to understanding work relationships as lived, relational experiences.

With these values in mind, this article carries out three objectives. First, it offers brief overviews of workplace relationship research in general as well as the directions qualitative research has taken in studying workplace relationships. To accomplish this, the article first briefly reviews the past decade of workplace relationship research in general as well as empirical work specifically using qualitative methods for understanding workplace relationships. Second, drawing from these overviews, the article offers and reinvigorates three generative directions for qualitative workplace relationship research: dyadic and multiadic approaches, thoughtful engagement with artifacts, documents, bodies, and other materials, and the use of topic-centered approaches to qualitative inquiry. Attention is given to topic-centered research, which can—although need not necessarily—serve as an important corrective when dominant methodological paradigms risk erasing the epistemological commitments of interpretive and critical qualitative work. When grounded in participant-centered meaning-making rather than theory-driven deduction, topic-centered approaches offer a powerful means of examining emergent workplace phenomena, vernacular practices, and culturally salient terms in ways that preserve qualitative values.

Finally, the article addresses key considerations for doing and evaluating quality qualitative workplace relationship research. By engaging Tracy’s (2010) big-tent criteria alongside a list of practical considerations for reporting qualitative studies, this section emphasizes rigor without reducing qualitative inquiry to procedural checklists. Taken together, this article is intended to serve as both a primer for conducting rigorous qualitative research on workplace relationships and a source of inspiration for scholars seeking new directions in this vibrant and consequential area of study.

### 1.1. Current Conceptualizations and Directions of Workplace Relationship Research

Over the past decade, workplace relationship research has continued to expand across communication, management, psychology, and public administration, reflecting a broad consensus that the quality of work itself is deeply intertwined with the quality of relationships among organizational members. As Sias and Shin (2019) argue, “the quality of an organization is virtually inextricable from the quality of relationships among the people who comprise the organization” (p. 187), a claim that has become foundational across contemporary workplace scholarship. Research during this period has increasingly emphasized communication as the primary mechanism through which workplace relationships are formed, enacted, evaluated, and linked to individual and organizational outcomes.

#### 1.1.1. Dominant Relationship Types and Conceptual Frameworks

A central feature of recent workplace relationship research is its continued organization around key relational types. Synthesizing decades of organizational communication scholarship, Sias and Shin (2019) identify four primary categories of workplace relationships that dominate the literature: supervisor–subordinate relationships, peer relationships, workplace friendships, and romantic workplace relationships. Among these, supervisor–subordinate relationships—often examined through *Leader–Member Exchange* (LMX) theory—remain the most extensively studied. Research grounded in LMX conceptualizes relationship quality as varying across dyads, with high-quality relationships characterized by trust, mutual respect, and communicative openness, and low-quality relationships associated with formality, distance, and reduced support.

Peer relationships have received growing attention alongside supervisor–subordinate dynamics, particularly as organizations increasingly rely on teamwork, collaboration, and interdependence. Peer relationships are often conceptualized as relatively symmetrical relationships that provide emotional support, informational resources, feedback, and identity validation. Workplace friendships, which may develop from peer relationships but are marked by greater personal disclosure and affective connection, have similarly been shown to influence job satisfaction, organizational commitment, and well-being. Romantic workplace relationships, while less common, remain a focal point due to their ethical, legal, and organizational implications, as well as their capacity to blur work–life boundaries.

Across these relationship types, contemporary scholarship consistently treats communication as both constitutive and instrumental. Communication is conceptualized not only as a means of exchanging information or coordinating tasks, but also as the process through which relational meanings, expectations, and identities are negotiated over time. As Mikkola and Nykänen (2019) note, workplace relationships are “fundamental systems of workplace communication” (p. 15), simultaneously shaping and being shaped by organizational culture, norms, and power relations.

#### 1.1.2. The Rise of Relationship Quality and Outcome-Oriented Models

Another prominent direction in workplace relationship research over the past decade has been the emphasis on *relationship quality* as a central explanatory construct. Rather than examining the mere presence or absence of relationships, many studies now focus on the perceived quality of workplace relationships and their associations with key outcomes such as commitment, performance, well-being, stress, and social impact.

For example, Caillier’s (2017) work in public organizations conceptualizes high-quality workplace relationships as positive, short-term dyadic interactions characterized by mutual engagement, vitality, and trust. Drawing from organizational behavior and positive organizational scholarship, this line of research links high-quality relationships to increased organizational commitment, perceptions of social impact, and—more unexpectedly—complex relationships with stress and exhaustion. Such findings underscore the multifaceted nature of workplace relationships, suggesting that relational closeness may simultaneously buffer stress while intensifying emotional investment in work.

Similarly, large-scale reviews and survey-based studies across management and applied business contexts continue to document associations between effective communication, supportive relationships, employee satisfaction, morale, and organizational performance. Although these studies vary in theoretical grounding and methodological rigor, they collectively reinforce the assumption that workplace relationships are critical resources for both individuals and organizations (Haq & Faizan, 2023).

#### 1.1.3. Personal Workplace Relationships and Work–Life Intersections

A particularly influential development in recent conceptual work is the articulation of *personal workplace relationships* (PWRs) as a distinct category of relational experience. Chory and Horan (2023) define PWRs as voluntary, informal, and emotionally significant relationships between organizational members that span work and life domains. This conceptualization explicitly foregrounds the personal dimensions of workplace relating, challenging models that treat workplace relationships as purely role-bound or instrumental.

Importantly, the framework for PWRs situates workplace relationships at the intersection of organizational and personal life, emphasizing how relational communication frequently crosses formal boundaries. By integrating perspectives from interpersonal communication, organizational communication, psychology, and management, this work highlights the interdisciplinary nature of workplace relationship research while also exposing persistent silos across fields. Chory and Horan argue that despite decades of scholarship, workplace relationships that blend personal and professional dimensions remain under-theorized and unevenly integrated into broader organizational research agendas.

In addition to work emphasizing supportive, high-quality, and voluntary workplace relationships, recent scholarship has increasingly attended to strained, harmful, and ethically complex relational dynamics at work. Research on workplace deviance, bullying, and dishonesty has expanded conceptualizations of workplace relationships by examining how relational processes can produce harm, exclusion, and identity threat rather than connection or support. Systematic reviews of workplace culture and deviant behavior demonstrate that relational norms, shared meanings, and communicative expectations embedded within organizational cultures strongly influence whether behaviors such as harassment, aggression, withdrawal, and rule violations are normalized or resisted (Bujang et al., 2024). Importantly, this work suggests that deviant behavior is not solely an individual pathology but is relationally and culturally produced through ongoing interaction, social exchange, and sensemaking processes.

Relatedly, extensive reviews of workplace bullying conceptualize bullying as an escalating relational process marked by repetition, power asymmetries, and communicative hostility that unfolds over time (Rai & Agarwal, 2016). Although often studied as an outcome or stressor, bullying research underscores that workplace relationships can become sites of systematic identity degradation, stigmatization, and social exclusion, highlighting the need to examine relational processes rather than isolated behaviors or traits. Complementing this work, identity-based models of workplace dishonesty conceptualize lying not simply as unethical individual action but as a socially motivated relational practice used to manage personal, relational, and collective identity threats within ongoing workplace relationships (Leavitt & Sluss, 2015). Together, these perspectives expand workplace relationship research beyond positive or functional ties, demonstrating that relationships also constitute sites where power, morality, identity, and harm are communicatively negotiated.

Another influential stream of research shaping contemporary workplace relationship scholarship centers on emotion, affect, and regulation as inherently relational processes. Reviews of emotion in organizations emphasize that emotions are not simple internal states but unfold through interpersonal interaction, communicative expression, and social interpretation (Ashkanasy & Dorris, 2017; Elfenbein, 2023). From this perspective, workplace relationships are primary sites where emotions are elicited, regulated, shared, and made meaningful. Interactions with supervisors, peers, and clients are among the most frequent emotional events at work, linking relational communication directly to experiences of stress, trust, belonging, conflict, and well-being. Importantly, this literature challenges simplistic distinctions between “positive” and “negative” emotions, instead emphasizing their social and functional roles in coordinating behavior, maintaining relationships, and negotiating power and hierarchy within organizations.

Building on this foundation, research on interpersonal emotion regulation (IER) explicitly conceptualizes emotion regulation as a relational accomplishment rather than an exclusively individual task. Troth et al. (2018) document how employees routinely attempt to influence not only their own emotional experiences but also those of others through communicative strategies such as reassurance, suppression, reframing, humor, and emotional attunement. These processes are deeply embedded in workplace relationships, shaping perceptions of support, friendship, leadership, and relational quality over time. Complementary work on mindfulness at work further underscores the relational implications of emotional awareness and regulation. Although often framed as an individual capacity, mindfulness has been linked to improved communication, empathy, conflict management, and relationship quality in workplace settings (Good et al., 2016). Taken together, these bodies of research position emotions, regulation, and attentional practices as central mechanisms through which workplace relationships are enacted and sustained; while also revealing a tendency toward variable-centered, outcome-oriented models that abstract emotional processes from their lived, interactional contexts.

A further expansion of workplace relationship research over the past decade reflects growing attention to the technological and ecological contexts in which workplace relationships are now embedded. Rather than treating communication technologies as neutral channels, contemporary scholarship increasingly conceptualizes workplaces as complex communication ecologies composed of multiple, overlapping media that jointly shape relational interaction (Turner et al., 2010). Employees routinely assemble and navigate constellations of face-to-face interaction, email, instant messaging, enterprise social media, and collaborative platforms, with each medium affording distinct relational possibilities and constraints. Research on digital communication environments emphasizes that these platforms simultaneously enable connection, visibility, and coordination while also introducing new relational tensions related to surveillance, accountability, responsiveness norms, and boundary management (Sivunen & Laitinen, 2019). From this perspective, workplace relationships are not merely enacted *through* technology but are increasingly *organized by* it, as relational expectations, availability, and identity performances become shaped by persistent, searchable, and often asynchronous interactional traces.

Related work on technostress, connectivity, and work–life interference further complicates contemporary conceptualizations of workplace relationships by foregrounding the emotional and relational costs of constant communication (Stich et al., 2018). Research demonstrates that communication technologies can intensify relational strain through overload, interruption, cyberdeviance, and the erosion of temporal boundaries between work and nonwork relationships. These dynamics are relational rather than purely individual, emerging through shared norms of responsiveness, implicit expectations about availability, and interactional practices that normalize constant access to others. At the same time, forward-looking scholarship on workplace communication highlights how digitalization, artificial intelligence, and flexible employment arrangements are reshaping the very conditions under which workplace relationships form and endure (Valo & Sivunen, 2019). As work becomes increasingly project-based, distributed, and mediated, interpersonal communication competence and relational sensemaking are positioned as central resources for sustaining collaboration, trust, and identification in the absence of stable organizational structures. Collectively, these strands of research extend workplace relationship scholarship beyond discrete relationship types or outcomes, underscoring the need to examine how evolving communicative environments, technologies, and futures of work fundamentally reconfigure relational processes at work.

#### 1.1.4. Communication as Constitutive of Workplace Relationships

Across these diverse conceptualizations, a shared assumption emerges: workplace relationships are communicatively constituted. Whether examined through LMX, social exchange, relational dialectics, or work–life intersection frameworks, contemporary scholarship consistently positions communication as the site where relational meanings are produced and negotiated. Mikkola and Nykänen (2019) emphasize that task-related, relational, and identity-oriented communication are inseparable in workplace interaction, with employees simultaneously coordinating work, managing impressions, and constructing relational expectations.

At the same time, much of this work—particularly in applied and management-oriented journals—has favored variable-centered, outcome-driven models that privilege measurement, prediction, and generalizability. While these approaches have yielded valuable insights into the consequences of workplace relationships, they often leave underexamined the lived, situated, and meaning-centered processes through which relationships are experienced and understood by organizational members themselves.

Taken together, workplace relationship research over the past decade reflects a robust and interdisciplinary field grounded in the recognition that relationships matter deeply in work contexts. Scholars have clarified key relationship types, documented links between relational quality and important outcomes, and increasingly acknowledged the personal dimensions of workplace relating. At the same time, the conceptual landscape remains shaped largely by models that prioritize roles, outcomes, and generalized patterns. As the next section demonstrates, qualitative workplace relationship research offers a complementary—and often underutilized—set of tools for examining how workplace relationships are communicatively constituted in everyday practice and across time.

### 1.2. Recent Research Developments in Qualitative Workplace Relationship Research

Over the past decade, qualitative research on workplace relationships has expanded in both scope and sophistication, offering nuanced accounts of how relational life at work is experienced, interpreted, and negotiated across contexts. Rather than centering solely on predefined relationship types or outcome variables, many recent qualitative studies foreground the lived realities of employees as they navigate support, conflict, identity, ethics, and well-being in organizational settings. This body of work demonstrates the value of qualitative approaches for capturing relational processes as they unfold over time, across roles, and within broader cultural and institutional ecologies. Collectively, these studies illustrate how workplace relationships are not simple, static entities, but instead are dynamic accomplishments shaped through communication, power, emotion, and meaning-making.

#### 1.2.1. Well-Being, Resilience, and Relational Support in Occupations

One prominent cluster of qualitative workplace relationship research focuses on well-being, resilience, and relational support, particularly in high-stress and care-intensive occupations. Qualitative studies in healthcare settings are especially illustrative. For example, Foster et al. (2018) and McDonald et al. (2016) both demonstrate how nurses and midwives experience resilience not as an individual disposition but as a relational accomplishment sustained through coworker support, mentoring, communicative openness, and perceived organizational care. Similarly, Ahmad et al. (2024) show how university teachers interpret workplace stress not as simple workload accumulation but as embedded in relational climates shaped by working conditions, promotion processes, and organizational atmosphere.

Recent phenomenological research further complicates simplistic accounts of “positive” relational systems. Fiaz and Qureshi (2023) illustrate how positive workplace relational systems function as dynamic relational networks that produce both constructive outcomes (e.g., enhanced well-being, generosity, performance) and unintended consequences such as perceptions of organizational politics. Their findings underscore that relational systems are not uniformly beneficial; rather, their outcomes depend on duration, quality, and perceived need satisfaction. Together, these studies position workplace well-being as communicatively and relationally constituted rather than individually possessed.

Qualitative research on communication-intensive work environments likewise reveals how relational conditions—rather than formal policies alone—shape employees’ capacity to cope with stressors. Gyllensten et al.’s (2023) study of noise in healthcare and preschool settings shows how sound environments saturated with talk, alarms, and interaction become relational stressors that affect trust, concentration, and emotional regulation. These studies underscore how workplace relationships are experienced through embodied, sensory, and emotional dimensions that are particularly well suited to qualitative investigation.

#### 1.2.2. Conflict, Harm, and Relational Breakdown in Workplace Relationships

A second cluster of qualitative research examines conflict, harm, and relational breakdown, challenging overly functionalist depictions of workplace relationships. Studies of workplace bullying, adversity, and communication conflict illustrate how relational harm emerges through repeated interaction, power asymmetries, and organizational cultures that normalize silence or aggression. Qualitative syntheses of bullying among nurses reveal patterns of lateral violence, institutional complicity, and identity erosion that unfold over time rather than as isolated incidents (Lee et al., 2022).

More recent qualitative work further highlights the dynamic and interpretive dimensions of relational harm. Garant et al. (2025), drawing on phenomenological interviews with workplace mediators, demonstrate how uncivil interactions evolve over time through attribution processes shaped by personal norms. Their findings emphasize that perspective taking—and the willingness to express vulnerability—can interrupt cycles of escalating incivility and restore trust. In contrast, the absence of such relational work often solidifies breakdown.

Similarly, Siddiqui and Iqbal (2024) explore toxic leadership in higher education institutions, showing how destructive leader–member exchanges are sustained through cultural and structural conditions, not simply individual pathology. Linvill (2025) further extends this line of inquiry by examining how targets of destructive workplace behaviors negotiate tensions between organizational identification and disidentification, often separating the organization from the perpetrator or anchoring identification in alternative relational ties.

Complementing this work, qualitative studies of communication conflict in organizational settings identify poor communication, mistrust, and unmet relational expectations as central antecedents of conflict, with outcomes including withdrawal, stress, and damaged relationships (Deep et al., 2016). Together, these studies demonstrate how qualitative approaches capture conflict not as episodic dysfunction but as an evolving relational process embedded in everyday interaction and institutional norms.

#### 1.2.3. Workplace Relationships and Everyday Practices

Recent qualitative research has turned attention to everyday relational practices that subtly shape workplace relationships, including gossip, technology use, privacy management, and embodied communication. Greenslade-Yeats et al.’s (2025) qualitative study of workplace gossip demonstrates how gossip operates as a relational sensemaking practice that can both strengthen and undermine interpersonal relationships, depending on how it is framed, shared, and interpreted. Similarly, Martinsson and Thomée’s (2025) qualitative exploration of coworker phubbing reveals how smartphone use during interaction is experienced relationally—as signaling disrespect, disengagement, or shifting priorities—rather than solely as a personal habit.

Qualitative scholarship has also examined how employees navigate identity-related disclosure and concealment in everyday interaction. Tomas et al. (2022), through a qualitative meta-synthesis of individuals with non-visible disabilities, illuminate the complex decision-making processes surrounding workplace disclosure, including timing, logistics, and anticipatory interpretations of others’ reactions. Steimel (2021), drawing on Communication Privacy Management theory, shows how women experiencing pregnancy loss negotiate privacy boundaries at work in light of colleagues’ responses and organizational taboos.

These studies exemplify a qualitative sensitivity to micro-level communicative practices through which relational norms, boundaries, and moral judgments are negotiated in real time. By foregrounding lived experience and interactional nuance, such research reveals how everyday practices become consequential sites of relational meaning-making.

#### 1.2.4. Trust, Inclusion, and Relational Meaning in Contemporary Work Relationships

Qualitative workplace relationship research has also made significant contributions to understanding trust, inclusion, and relational meaning in contemporary and globally diverse work arrangements. Fischer and Walker’s (2022) qualitative exploration of trust in blended virtual and face-to-face workplaces demonstrates that trust is constructed through communicative behaviors such as transparency, responsiveness, and relational exposure, rather than through abstract attitudes alone. Similarly, qualitative studies of inclusion show how employees interpret belonging and exclusion through everyday interactions involving voice, recognition, and access to information (Rezai et al., 2023).

Research on expatriate and cross-cultural professionals further underscores the relational foundations of workplace adjustment. Chudnovskaya (2022) finds that foreign professionals’ success in U.S. workplaces hinges not only on individual competence but on communicative relationships, cross-cultural adjustment, and the cultivation of relational trust within organizational systems.

Feminist qualitative scholarship likewise highlights the relational construction of inclusion and exclusion. Lin et al. (2025) introduce the concept of performativity dynamics to explain how women in public relations navigate gendered peer pressure, strategically managing identity performances within relational and organizational constraints. Zou et al. (2026), through critical metaphor analysis, reveal how women’s embodied experiences of dysmenorrhea intersect with workplace expectations, gender hierarchies, and relational power, positioning the body itself as a site of relational negotiation and institutional meaning.

Collectively, these studies illustrate how qualitative research captures the relational labor involved in sustaining trust, inclusion, identification, and coordination under changing organizational conditions. Rather than treating workplace relationships as static ties, this body of work foregrounds the communicative processes through which relational meaning is continually constituted and contested.

### 1.3. Methodological Commitments Across Research Areas

Across these diverse topical areas, recent qualitative workplace relationship research is marked by several shared methodological commitments. Many studies adopt inductive, phenomenological, or constructivist approaches that privilege participants’ sensemaking while remaining attentive to theory development. Others employ qualitative synthesis techniques to integrate findings across contexts, revealing recurring relational patterns while still honoring institutional and cultural specificity. Importantly, these studies reflect a range of interpretive, critical, and applied traditions, unified less by method per se than by a shared commitment to understanding workplace relationships as communicatively constituted, culturally embedded, and ethically consequential. Taken together, this body of work demonstrates both the richness and diversity of contemporary qualitative workplace relationship scholarship and sets the stage for identifying generative directions that move beyond cataloging relationship types—although such work is valuable, too—and toward deeper engagement with relational processes, materials, and topics.

#### 1.3.1. Looking Ahead: Embracing the Benefits of Qualitative Workplace Relationship Studies

The qualitative studies reviewed in the preceding section demonstrate both the vitality of contemporary workplace relationship research and the ongoing opportunities to deepen how such relationships are conceptualized and studied. Taken together, this work illustrates that workplace relationships are not only attitudinal states or role-based interactions, but lived, communicative accomplishments shaped through interaction, material conditions, and shared meanings. At the same time, much qualitative workplace research continues to rely on individual perspectives, abstracted contexts, or theory-driven frames that can inadvertently limit the relational, contextual, and interpretive potential of qualitative inquiry.

Looking ahead, qualitative workplace relationship research is especially well positioned to advance the field by embracing methodological approaches that foreground relationships as the primary analytic unit. This requires moving beyond studies that treat relationships as outcomes or background variables and toward designs that capture how relationships are jointly constituted across perspectives, mediated by objects and environments, and understood through participants’ own terms. Such an orientation aligns with interpretive and constitutive traditions in communication research, while also responding to the complexities of contemporary workplaces characterized by technological mediation, shifting norms, and evolving relational vocabularies.

The following sections outline three generative directions for qualitative workplace relationship research that build on existing empirical traditions while pushing the field forward. First, dyadic and multiadic approaches are introduced as a means of examining relationships across interacting perspectives rather than through isolated accounts. Second, sociomaterial analyses are advanced to highlight how objects, documents, bodies, and spaces participate in the constitution of workplace relationships. Third, topic-centered qualitative approaches are proposed as a way to preserve the epistemological strengths of qualitative inquiry by theorizing emergent workplace phenomena from participants’ lived experiences. Together, these directions offer a cohesive methodological vision for studying workplace relationships as dynamic, situated, and meaning-centered processes.

#### 1.3.2. Dyadic and Multiadic Approaches to Workplace Relationship Research

Despite the growing sophistication of qualitative workplace relationship research, much of this work continues to rely on the accounts of single participants. Although individual interviews offer valuable insight into personal sensemaking, they are limited in their capacity to capture relationships as communicatively constituted phenomena. Relationships are, by definition, co-created through interaction, negotiation, and mutual orientation (see Manning, 2020). When qualitative workplace studies rely exclusively on one person’s account of a relationship, they risk reifying relationships as internal states rather than examining them as dynamic, relational processes that unfold between people and within particular organizational contexts. Dyadic and multiadic qualitative approaches offer a powerful means of addressing this limitation by centering relational interaction, divergence, and convergence as objects of analysis rather than methodological complications.

Drawing from qualitative traditions in interpersonal and family studies, Manning and Kunkel (2015) argue that dyadic approaches allow researchers to observe how meaning is constructed across perspectives rather than inferred from a single narrative. Rather than treating differences between participants’ accounts as problems of validity or reliability, interpretive dyadic research views such differences as analytically productive. Divergent narratives illuminate how relationships are constituted through competing discourses, asymmetries of power, affective investments, and contextual positioning. Applied to workplace research, dyadic designs make it possible to examine how supervisors and subordinates, coworkers, mentors and protégés, or collaborators jointly—and sometimes conflictually—construct relational realities such as trust, support, conflict, or inclusion.

Dyadic approaches also invite methodological designs that move beyond a single interview format. Manning and Kunkel (2015) outline multiple configurations—including individual interviews with each relational partner, joint interviews, and combinations of both—that allow researchers to trace how meanings shift across interactional contexts. In workplace settings, for example, an employee’s private account of a supervisor relationship may differ substantially from how that relationship is described—or enacted—when both parties are present, or when the relationship is discussed in a team context. Such designs enable researchers to examine not only what people say about workplace relationships, but how relational meanings are managed, muted, amplified, or contested across settings.

Extending beyond dyads, *multiadic approaches* are especially well suited to contemporary workplaces, where relationships rarely exist in isolation. Multiadic analysis attends to how multiple relational configurations—such as peer groups, teams, departments, or cross-functional units—intersect and influence one another. As Manning (2013) suggests, multiadic analysis allows researchers to trace how discourses circulate across individual, dyadic, and group contexts, revealing how meanings introduced in one relational space are reinforced, transformed, or silenced in another. In workplace research, this approach makes it possible to examine how organizational norms, policies, and informal cultures shape relationships differently across relational configurations, rather than assuming a singular relational reality.

Importantly, dyadic and multiadic approaches also create space for incorporating the workplace itself more fully into qualitative analysis. When researchers attend to how relationships are enacted across interactions and relational configurations, organizational artifacts, policies, technologies, and physical environments often emerge as active participants in relational sensemaking. Differences across interviews may hinge on spatial arrangements, communication technologies, scheduling practices, performance metrics, or institutional discourses that structure interaction. In this way, dyadic and multiadic qualitative designs invite sustained attention to the sociomaterial conditions under which workplace relationships are constituted and maintained—conditions that are frequently backgrounded in single-participant studies. Table 1 illustrates how these approaches may be operationalized in workplace interviewing research.

Taken together, dyadic and multiadic approaches offer qualitative workplace relationship researchers a means of studying relationships as lived, interactional, and situated phenomena. These approaches do not replace individual interviews; rather, they extend them by foregrounding relational complexity, contextual variability, and meaning-making across perspectives. By drawing multiple participants into the same relational analytic frame—and by attending to how workplace contexts shape interaction—dyadic and multiadic qualitative research provides a methodological bridge between interpersonal process and organizational environment. The next section builds on this bridge by turning explicitly to sociomaterial perspectives that further illuminate how relationships are constituted through objects, spaces, technologies, and institutional arrangements at work.

#### 1.3.3. Analysis of Objects, Documents, Bodies, and Other Materials

Qualitative workplace relationship research has increasingly recognized that relationships are not constituted solely through talk or subjective interpretation, but through ongoing engagement with objects, documents, spaces, bodies, and technologies that both enable and constrain interaction. Sociomaterial perspectives challenge the tendency to treat materiality as a background context for social processes, instead positioning material forms as integral to how relationships are enacted and experienced. From a communication-as-constitutive-of-organization (CCO) perspective, materiality matters insofar as it becomes consequential in interaction and made present, absent, or authoritative through communicative practice (Arnaud & Fauré, 2019; Cooren, 2020). Analyzing material forms, then, is not an analytic add-on but a necessary extension of relational inquiry, particularly in workplaces where documents, technologies, and spaces routinely mediate relational expectations and power.

Much sociomaterial research in organizational studies has focused on workplace-centered materials such as technologies, policies, documents, and physical layouts to understand organizing processes. Studies of artifacts—including office design, tools, visual representations, and workflows—demonstrate how material arrangements shape authority, coordination, and identity while affording or constraining interaction (Bechky, 2008; Våland & Georg, 2014). Document analysis, in particular, has shown how texts such as policies, emails, performance evaluations, and procedural manuals function as active participants in organizational life rather than neutral records of intent (Bowen, 2009). These materials do relational work: they authorize action, distribute accountability, and stabilize expectations across time and space. Yet, in much of this literature, relationships are treated as downstream effects of material systems rather than as primary sites where materiality becomes meaningful.

A relationship-centered sociomaterial approach shifts the analytic focus toward how material forms participate in the constitution of specific workplace relationships. Rather than asking how documents or technologies shape “the organization,” this approach asks how they mediate trust, conflict, support, surveillance, and relational identity between people. For example, shared documents may function as sites of negotiation or contestation within supervisor–subordinate relationships; scheduling software or messaging platforms may recalibrate expectations for responsiveness and availability among coworkers; and spatial arrangements may shape privacy, affiliation, or emotional safety within teams. Drawing on relational communication scholarship, this orientation treats objects and texts as relational artifacts: resources through which relationships are remembered, maintained, constrained, or repaired (Manning, 2016). In this sense, sociomaterial analysis complements dyadic and multiadic qualitative designs by situating interaction within the material environments that give it consequence.

Expanding sociomaterial analysis also invites greater attention to bodies, affect, and embodiment as material dimensions of workplace relationships. Bodies are not neutral carriers of communication but sites where professionalism, vulnerability, difference, and power are enacted and interpreted. Physical proximity, posture, movement, dress, and sensory conditions—such as noise, lighting, or temperature—shape how relational boundaries are drawn and experienced (see, for example, Ellingson, 2017). Qualitative approaches attentive to embodiment allow researchers to examine how relational meanings are negotiated through presence, absence, and affective display, extending sociomaterial analysis beyond objects and texts alone. Actor–network-informed approaches further underscore that relational processes may be sustained through nonhuman actants even when direct interaction is limited or strained, highlighting how relationships persist through networks of human and material association rather than continuous talk alone (e.g., Allen & Allen, 2021).

Taken together, sociomaterial approaches offer qualitative workplace relationship researchers a means of examining how relationships are constituted through entanglements of people, practices, and material forms. Importantly, a relationship-centered sociomaterial orientation resists reducing material analysis to organizational infrastructure while also avoiding a return to purely symbolic or discursive models of relating. By analyzing objects, documents, bodies, and spaces as relationally consequential rather than simply organizationally functional, researchers can develop richer accounts of how workplace relationships are lived, negotiated, and sustained. The following section builds on this orientation by turning to topic-centered qualitative approaches that foreground relationships as the primary analytic concern, while also remaining attentive to the material and contextual conditions under which they unfold.

#### 1.3.4. Using Topic-Centered Approaches

A recurring challenge in qualitative workplace relationship research is the tendency for studies to adopt post-positivist assumptions while using qualitative data, a move that can subtly erode the interpretive strengths of qualitative inquiry. As Braithwaite et al. (2014) observe, qualitative scholarship is often evaluated according to criteria better suited to variable-centered, deductive research, including expectations for hypothesis testing, prediction, and measurement. When these assumptions are imported into qualitative designs, researchers might feel compelled to frame studies around predefined constructs, narrow research questions, or theory-driven models that constrain what participants can reveal. These concerns are not critiques of quantitative or post-positivist workplace research, but of the misapplication of their evaluative logics to qualitative inquiry. Topic-centered approaches offer an alternative by allowing researchers to examine emergent workplace phenomena on their own terms, privileging participant meanings and vernacular understandings rather than forcing data into preexisting conceptual or theoretical boxes.

Tracy’s (2012) critique of deductive writing logics for inductive qualitative research clarifies why this drift is particularly problematic. When qualitative studies are written as though they were designed deductively—complete with a priori research questions, tightly bounded constructs, and confirmatory logics—they risk misrepresenting the actual analytic process and narrowing theoretical imagination. In workplace relationship research, this can result in studies that appear qualitative in method but post-positivist in spirit, emphasizing categorization over sensemaking and confirmation over discovery. Topic-centered qualitative research resists this tendency by beginning with phenomena that matter to participants—such as emerging workplace practices, relational labels, or shared concerns—and allowing theory to emerge through iterative engagement with data rather than serving as a template imposed in advance.

Importantly, topic-centered approaches do not reject theory; rather, they reconceptualize theorizing as an outcome of sustained engagement with participants’ lived experiences. What if, instead of testing whether a concept “works,” qualitative researchers developed grounded or interpretive theories *of* workplace relationship topics themselves? Concepts such as “quiet quitting,” “backchannel communication,” “work spouse,” or “emotional availability at work” circulate widely in organizational discourse yet remain undertheorized from participants’ perspectives. Topic-centered qualitative studies can illuminate how such terms are understood, contested, and enacted in everyday relational practice, revealing nuances that are often flattened in variable-centered research. In this sense, topic-centered approaches expand theoretical possibilities by taking seriously the knowledge already embedded in workplace vernacular.

The construct of *job crafting*, for example, offers a useful illustration of how topic-centered qualitative inquiry can complement—and complicate—existing theory. Quantitative research, including Rudolph et al.’s (2017) meta-analysis, has established job crafting as a proactive behavior linked to job demands, resources, and outcomes. Yet qualitative, topic-centered studies of job crafting reveal how employees experience and interpret these practices relationally—through negotiations with coworkers, implicit permission structures, and shared understandings of what kinds of change are acceptable. From a qualitative standpoint, job crafting is not simply an individual behavior but a relational accomplishment embedded in workplace norms, power relations, and identity work. Topic-centered qualitative approaches thus enrich existing models by showing how ostensibly individual practices are relationally constituted and unevenly available across contexts.

Taken together, topic-centered approaches reaffirm the value of qualitative workplace relationship research as a site of discovery, theorizing, and critique. By resisting the pressure to perform post-positivist rigor “in drag,” qualitative researchers can remain attentive to participants’ meanings while still producing conceptually generative scholarship. Topic-centered studies allow researchers to trace how workplace relationship phenomena emerge, evolve, and take on significance across contexts, offering insights that are difficult—if not impossible—to capture through deductive designs alone.

## 2. Design Considerations in Expanding Qualitative Workplace Relationship Research

As qualitative workplace relationship research increasingly embraces dyadic, multiadic, sociomaterial, and topic-centered approaches, several design considerations warrant careful attention. These are not methodological flaws but practical and ethical complexities that shape how such studies unfold in real organizational contexts.

### 2.1. Dyadic and Multiadic Designs

Dyadic and multiadic interview designs offer significant analytic richness, yet they also require substantial logistical coordination. Scheduling multiple participants—particularly across hierarchical levels or dispersed teams—can be time-intensive and may require navigating competing organizational priorities. Coordinating interview sequencing (e.g., individual before joint) adds another layer of planning that directly shapes the data generated.

Confidentiality management becomes especially complex in these designs. Researchers must carefully delineate what information, if any, may be referenced across interviews, and participants must be reassured that private disclosures will not inadvertently surface in joint settings. This is particularly critical when studying sensitive topics such as incivility, leadership toxicity, disclosure, or conflict.

In these cases, potential for relational fallout is also a consideration. Joint interviews or comparative analyses may surface tensions, contradictions, or grievances that extend beyond the research setting. Although researchers cannot control relational outcomes, they must anticipate and mitigate the possibility that participation could strain workplace relationships, especially in contexts marked by power asymmetries.

Finally, dyadic and multiadic designs introduce analytic complexity. Researchers are not simply coding individual transcripts but tracing overlap, contradiction, silence, discursive shifts, and relational positioning across interviews. Such analyses require theoretical clarity, disciplined data management, and a willingness to engage interpretively with competing accounts rather than collapsing them into a singular narrative.

### 2.2. Sociomaterial Analysis

Approaches that foreground sociomaterial conditions—artifacts, technologies, spatial arrangements, institutional discourses—introduce additional considerations. Access to communication artifacts may be restricted due to privacy policies, proprietary concerns, or organizational sensitivities. Emails, performance documents, messaging platforms, architectural layouts, and policy manuals often require explicit permission, and access may be uneven across participants.

Ethical boundaries must also be clearly defined. The inclusion of artifacts or technologies can blur distinctions between public and private communication, especially when analyzing digital interactions. Researchers must carefully consider consent, anonymization, and the potential for re-identification in highly networked workplace environments.

Sociomaterial approaches also increase the risk of data overload. When interviews are supplemented by documents, digital traces, or environmental observations, the volume and heterogeneity of data can become overwhelming. Clear analytic boundaries and theoretically guided sampling of artifacts are essential.

Finally, organizational gatekeeping can shape what becomes visible. Access to spaces, documents, or communication platforms may be filtered through management or institutional review processes, potentially influencing both participation and representation. Researchers must remain reflexive about how access itself structures the data produced.

### 2.3. Topic-Centered Designs

Topic-centered qualitative designs—such as studies focused on stress, disclosure, leadership, inclusion, or workplace health—offer conceptual clarity but introduce their own tensions. One risk is over-fragmentation. When research is tightly organized around a single issue, broader relational processes may be inadvertently backgrounded, leading to analyses that isolate phenomena from their relational ecologies.

Relatedly, topic-centered designs may sacrifice relational depth. Focusing primarily on a phenomenon (e.g., incivility, gendered peer pressure, or disability disclosure) can shift attention away from the evolving dynamics of specific relationships over time.

Finally, researchers must balance sampling breadth with analytic coherence. Expanding participant pools across departments, roles, or institutions can illuminate variation, yet excessive diversification may dilute the interpretive coherence of findings. Careful sampling strategies and transparent analytic framing help maintain theoretical focus while preserving contextual nuance.

## 3. Developing Theory in Qualitative Workplace Relationship Research

Qualitative workplace relationship research is not only well-positioned to describe relational processes—it is also uniquely capable of developing, refining, and extending theory. While qualitative inquiry has sometimes been framed primarily as descriptive or exploratory, contemporary scholarship makes clear that theory-building is both a legitimate and necessary outcome of rigorous qualitative work.

Saldaña (2025) suggests that qualitative-informed theory may include at least one of six core properties: (1) a patterned relationship between two or more concepts; (2) prediction and/or management of action through propositional logic; (3) parameters and/or variation in empirical observations; (4) explanation of how and/or why something happens; (5) generalizability and/or transferability to related social contexts; and (6) insights and/or guidance for improving social life. These properties underscore that theory need not resemble formalized grand theory to be meaningful. Even localized qualitative studies can generate patterned conceptual relationships, explanatory mechanisms, or transferable insights that meaningfully contribute to disciplinary knowledge.

Importantly, theory can be embedded in qualitative research in multiple ways. Manning and Kunkel (2014b) argue that theory may be integrated into interview protocols or overall project designs—not as a rigid template that forces participants’ responses into preexisting categories, but rather as a sensitizing framework that creates opportunities for theoretical processes to emerge. The key is to avoid transforming theory into a “cookie cutter” that predetermines analytic outcomes. Instead, researchers should design studies that allow theory to be witnessed, refined, complicated, or even disrupted through interactional and observational data.

Recent methodological scholarship further emphasizes that theory in qualitative research should be explicit, flexible, and iterative. Nguyen et al. (2022) propose that researchers clearly articulate when and how theory enters the research process, whether during pre-design, data collection, analysis, or interpretation. Rather than treating theory as either absent or totalizing, qualitative researchers may “let theory in” and “let theory out” at strategic points in the study, depending on whether the aim is description, theory generation, or theory validation. This perspective reinforces the idea that theory application is not a binary decision but a dynamic methodological practice.

Similarly, Van Hulst and Visser (2025) advocate for abductive analysis as a productive mode of theory development. Abduction involves moving iteratively between empirical material and theoretical concepts, allowing surprising findings to provoke conceptual rethinking. In workplace relationship research, abductive reasoning is particularly generative: relational data often reveal tensions, contradictions, or unexpected alignments that neither purely inductive nor purely deductive approaches fully capture. Through abductive engagement, researchers can refine existing frameworks (e.g., LMX, personal workplace relationships, relational turbulence) or construct new theoretical explanations grounded in lived organizational experience.

Although grounded theory methodologies are explicitly designed for theory development, theory-building in qualitative workplace relationship research need not be confined to formal grounded theory designs. Depending on the data type and analytic orientation, researchers may develop a theory of a specific topic (e.g., relational repair after organizational restructuring), articulate a mid-range conceptual model (e.g., patterns of boundary negotiation in hybrid work environments), or advance a more comprehensive relational framework. Moreover, qualitative scholars can synthesize insights across multiple datasets—whether within a program of research or across collaborative projects—to generate broader theoretical propositions with greater transferability.

Qualitative data are especially powerful in providing nuance, scope, and depth to theoretical claims. Rich interactional accounts, narrative detail, and contextual sensitivity allow researchers to specify the conditions under which relational processes unfold. Such data illuminate parameters, variations, and mechanisms—precisely the features Saldaña identifies as central to theory. In this sense, qualitative research does not simply illustrate theory; it sharpens its explanatory capacity.

Finally, once theoretical patterns begin to emerge from qualitative analysis, theory itself can serve as a makeshift coding device (Manning & Kunkel, 2014b). That is, researchers may return to the data with emerging conceptual insights in mind, examining how these patterns recur, vary, or fail across cases. This iterative movement between data and theory strengthens analytic rigor while preserving openness to revision.

## 4. Exemplars of Qualitative Theory Development

Qualitative workplace relationship scholarship already offers strong exemplars of theory development that meet multiple properties of qualitative theory as articulated by Saldaña (2025). For instance, Dougherty et al.’s (2009) development of *Language Convergence/Meaning Divergence (LC/MD) theory* illustrates how patterned relationships between concepts can emerge inductively from rich qualitative data. Drawing on grounded theory analysis of workplace social–sexual communication, the authors identify recurring processes—language convergence, meaning divergence, meaning clusters, and the illusion of shared understanding—that together constitute a coherent explanatory framework. LC/MD does more than catalog discursive variation; it explains how and why misunderstandings persist even amid apparent symbolic agreement. In doing so, it demonstrates how qualitative analysis can generate a theory that is conceptually precise, explanatory in scope, and transferable across relational and organizational contexts.

Similarly, Tracy and Huffman’s (2017) discourse analysis of compassionate communication in a crisis setting provides an example of theory refinement through close analysis of interactional detail. By examining the communicative moves through which compassion was enacted in a high-stakes encounter, the authors extend existing conceptualizations of compassion beyond abstract components (e.g., recognizing, relating, acting) to articulate specific conversational practices—such as timing, mirroring, co-creating hope, and vulnerable self-disclosure—that enable compassion in resistant contexts. Their work illustrates how qualitative research can move from case-based analysis to theoretical elaboration, offering both explanatory insight and practical guidance for improving social life.

Together, these exemplars underscore that qualitative workplace relationship research is well positioned not only to generate grounded, localized theories, but also to refine, extend, and sometimes reconfigure broader theoretical traditions. When researchers attend carefully to patterned relationships, parameters of variation, explanatory mechanisms, and implications for action, qualitative inquiry becomes one of the most generative sites for theory development in communication studies.

### 4.1. Quality and “Rigor” in Qualitative Workplace Relationship Research

Beyond post-positivist/interpretive–critical tensions, questions of quality and rigor are unavoidable in qualitative workplace relationship research—particularly as it is an area of research that spans multiple disciplinary homes, epistemological traditions, and methodological commitments. Too often, however, such questions are framed through standards derived from post-positivist research traditions, leading to misunderstandings about what constitutes rigor in qualitative inquiry and how it should be evaluated. In the context of workplace relationship research—where meaning, power, emotion, and ethics are deeply entangled—equating rigor with procedural uniformity or “checklist compliance” risks flattening the very phenomena qualitative methods are designed to illuminate.

This section approaches quality in qualitative workplace relationship research as an interpretive, ethical, and relational accomplishment rather than a technical achievement. Drawing on Tracy’s (2010) influential “big-tent” criteria, the first subsection offers a flexible framework for thinking about qualitative quality that honors methodological pluralism while articulating shared aspirations for excellent qualitative work. Particular attention is given to how these criteria resonate with the relational, contextual, and often sensitive nature of workplace relationship research, as well as to Tracy’s longstanding contributions to studying work, relationships, emotion, and organizational life.

The second subsection turns to practical considerations for reporting qualitative workplace relationship research. Building from Tong et al.’s (2007) consolidated reporting criteria, it introduces a relationship-centered reporting framework designed to support transparency and ethical accountability without reducing qualitative rigor to mechanical compliance. Together, these two approaches—conceptual guidance through big-tent criteria and practical support through reflective reporting—offer complementary tools for advancing high-quality qualitative workplace relationship research without sacrificing its interpretive strengths.

### 4.2. “Big-Tent” Criteria for Quality Qualitative Workplace Relationship Research

Tracy’s (2010) “big-tent” framework for qualitative quality has become one of the most influential and widely cited responses to questions regarding qualitative research quality, offering a set of eight flexible criteria—worthy topic, rich rigor, sincerity, credibility, resonance, significant contribution, ethics, and meaningful coherence—that can be applied across qualitative traditions. Developed in response to the persistent misapplication of post-positivist standards to qualitative inquiry, the big-tent framework provides a shared vocabulary for articulating quality while resisting methodological orthodoxy. Importantly for workplace relationship scholars, Tracy’s work is deeply grounded in organizational and workplace contexts, including extensive qualitative research on bullying, compassion, emotion labor, work–life negotiation, and relational harm, making the framework especially attuned to the ethical, relational, and interpretive demands of studying work.

One of the central strengths of the big-tent model is its distinction between *qualitative ends* and *qualitative means*. Rather than prescribing specific methods or analytic techniques, the framework identifies common goals that high-quality qualitative research should strive toward, while leaving room for multiple epistemological paths to achieve them (Tracy & Hinrichs, 2017). This flexibility is particularly valuable for qualitative workplace relationship research, which spans interpretive, critical, applied, and practice-oriented traditions. For example, credibility may be achieved through thick description, multivocality, or prolonged engagement, while ethics may involve careful attention to relational vulnerability, power asymmetries, and the long-term consequences of representation. In workplace relationship studies—where participants’ identities, livelihoods, and interpersonal ties are often at stake—the big-tent criteria foreground ethics, sincerity, and resonance as core dimensions of quality rather than ancillary concerns.

At the same time, Tracy’s framework has generated productive dialog and critique within the qualitative community, underscoring its role as a living, reflexive model rather than a fixed evaluative checklist. Scholars have raised questions about claims of universality and emphasized the importance of situating quality judgments within particular epistemological and ethical traditions (Gordon & Patterson, 2013). Tracy herself has consistently responded to such critiques by reaffirming that the big-tent model is intended as a heuristic for dialog, pedagogy, and advocacy rather than as a mechanism of gatekeeping (Tracy, 2026). For qualitative workplace relationship researchers, the value of the big tent lies precisely in this openness: it allows scholars to articulate rigor without defaulting to post-positivist benchmarks, to defend interpretive and topic-centered work on its own terms, and to communicate the value of relationally grounded qualitative research to diverse scholarly audiences. In this sense, the big-tent criteria offer not only standards for evaluation, but also a capacious ethical and intellectual space in which qualitative workplace relationship research can continue to grow.

## 5. Reporting Qualitative Workplace Relationship Research: Some Considerations

Clear, ethical, and transparent reporting is essential to the advancement of qualitative workplace relationship research, particularly in a field that spans multiple disciplines, epistemological commitments, and methodological traditions. At the same time, scholars have long cautioned against treating reporting checklists as proxies for rigor rather than as reflective aids embedded within broader qualitative sensibilities. Barbour (2001) famously warned that the uncritical use of checklists risks allowing “the tail to wag the dog” (p. 1115), reducing qualitative inquiry to a series of technical fixes divorced from the interpretive, contextual, and relational logics that give it value. In workplace relationship research—where meaning, power, emotion, and ethics are often deeply intertwined—such reductionism is especially problematic.

Nevertheless, thoughtfully adapted reporting frameworks can serve important purposes when used reflexively rather than prescriptively. Tong et al.’s (2007) Consolidated Criteria for Reporting Qualitative Research (COREQ) was originally developed for health research to improve transparency in interview—and focus-group—based studies, not to standardize qualitative practice or impose methodological uniformity. When explicitly acknowledged as a reporting aid rather than a measure of methodological virtue, a checklist can support ethical accountability, facilitate peer review, and conserve valuable manuscript or presentation space by signaling that key dimensions of qualitative rigor have been considered. As Barbour (2001) and Tracy (2010) both emphasize, rigor emerges not from the mechanical application of procedures but from coherence among epistemology, methods, analysis, and claims.

Drawing inspiration from COREQ while reconfiguring its logic around relational inquiry, Table 2 presents the *Qualitative Relationship Reporting Framework* (QRRF-4) organized around four interrelated domains. First, *researcher positioning and relational access* includes disclosure of researchers’ disciplinary location, relationship to the workplace or participants, and reflexive consideration of power, role, and access. Given that workplace relationship studies often involve ongoing organizational ties or sensitive relational dynamics, transparency about positionality is particularly critical. Second, *relationship-centered study design* addresses how relationships—rather than solely individuals or organizations—are conceptualized, sampled, and bounded, including attention to dyads, networks, or relational histories. Third, *data generation and analytic process* includes information about interactional contexts, communicative practices, analytic strategies, and how relational meanings were traced across time, roles, or material settings. Finally, *ethical and representational considerations* foreground confidentiality, relational risk, anonymization strategies, and the ethical implications of representing workplace relationships that may involve conflict, harm, or vulnerability. Although developed within the context of workplace relationship scholarship, the QRRF-4 is designed for qualitative relationship research more broadly, wherever relationships constitute the primary analytic unit.

The framework is offered not as a required template but as a structured signal of reflexive engagement. In interdisciplinary publication contexts—where reviewers may bring divergent expectations about qualitative transparency—authors often face pressure to justify methodological decisions in limited space. A concise, relationship-centered reporting framework allows scholars to indicate that key dimensions of positionality, relational sampling, analytic logic, and ethical risk have been considered, even when detailed explication would displace substantive theoretical development. In this sense, the framework operates as a communicative resource: it makes visible the interpretive labor already embedded in qualitative workplace relationship research.

Importantly, this list of considerations is offered as an *affirmative disclosure tool*, not as a gatekeeping mechanism. Authors might note, for example, that “the Qualitative Relationship Reporting Framework (QRRF) was consulted and is available in supplemental materials,” thereby signaling ethical and methodological consideration without displacing substantive theorizing or analysis. Used in this way, a checklist can function as a professional signal of care and transparency rather than a substitute for qualitative judgment. As Tong et al. (2007) emphasize, reporting criteria are intended to enhance clarity, not to constrain methodological imagination. While inspired by established reporting guidance, the QRRF-4 reframes reporting around relationships as the primary analytic unit, thereby extending rather than replicating prior tools.

In sum, qualitative workplace relationship research benefits from reporting practices that balance transparency with epistemological humility. Checklists can support this balance when they are explicitly framed as reflective aids situated within broader qualitative commitments. When treated as guides rather than rules, and when explicitly oriented toward the relational complexities of workplace life, reporting frameworks can enhance scholarly dialog while preserving the interpretive richness that gives qualitative workplace relationship research its distinctive contribution.

## 6. Conclusions

This article is intended for a broad audience of scholars who engage, or wish to engage, in qualitative workplace relationship research. Early-career researchers may find it especially useful as a guide for designing studies that honor qualitative epistemologies while navigating interdisciplinary expectations. More established scholars—particularly those feeling constrained by familiar designs or theoretical ruts—might find inspiration here for rethinking how relationships, materials, and topics can be approached in new ways. The article is also written with reviewers, editors, and interdisciplinary research teams in mind, offering a shared language for evaluating qualitative relationship research without defaulting to post-positivist assumptions that often misrepresent its goals and strengths.

In practice, this article’s tools may be used in multiple ways. Researchers can draw on them when designing qualitative studies of workplace relationships, particularly when considering dyadic and multiadic designs, sociomaterial analyses, or topic-centered approaches. The article can also serve as a resource for evaluating rigor, offering flexible yet principled guidance grounded in Tracy’s (2010) big-tent criteria and the Qualitative Relationship Reporting Framework (QRRF-4). Although developed within the context of workplace scholarship, the QRRF-4 is intended as a broadly applicable reporting aid for qualitative relationship research wherever relationships constitute the primary analytic unit. In teaching contexts, this article can support graduate methods courses by illustrating how qualitative research operates across interpretive, applied, and critical traditions; and, candidly, it provides scholars with language and citations for responding to persistent misconceptions about qualitative rigor, theory development, and methodological contribution.

Across the article, I have argued that qualitative workplace relationship research is at its strongest when it treats relationships as communicatively constituted, materially situated, and meaning-centered phenomena. The generative directions outlined here—dyadic and multiadic designs, sociomaterial analysis, and topic-centered approaches—are not simple methodological alternatives but pathways toward deeper theoretical and practical insight. Ultimately, this article serves as an invitation to approach qualitative relationship research with both confidence and curiosity: *Confidence* in recognizing that qualitative methods already offer robust tools for understanding relational life on their own epistemological terms; *Curiosity* in remaining open to new designs, relational configurations, and material engagements that reflect the evolving realities of contemporary work and organizing. By embracing methodological pluralism without abandoning epistemological integrity, qualitative workplace relationship research can continue to generate insight, theory, and impact—illuminating the complex, fragile, and deeply human labor of relating at work.

## Figures and Tables

**Table 1:** Dyadic and multiadic interview approaches in workplace communication.

Interview Type	Ideal Use	Benefits for Workplace Communication Studies	Limitations for Workplace Communication Studies	Sources for More Information
**1. Individual Interviews**	Sensitive topics (e.g., incivility, harassment, disclosure, toxic leadership); when private sensemaking is central; when confidentiality is critical	Allows participants to share experiences without fear of reprisal; captures individual meaning-making, identity negotiation, and emotional processing; useful for studying power asymmetries	Lacks direct observation of interaction; relational dynamics must be inferred; potential gaps between reported and enacted communication	Forbat and Henderson (2003); Ummel and Achille (2016)
**2. Individual Interviews by Different Interviewers**	Highly sensitive organizational contexts; when participants may discuss interviews with one another; when minimizing cross-interview contamination is important	Reduces risk of participants feeling information will be shared across interviews; helps maintain perceived neutrality in conflict-laden settings	Resource-intensive; interviewer style differences may influence data; coordination challenges; less continuity in probing across interviews	Hellstrom et al. (2005)
**3. Joint Interviews**	When observing live communicative interaction is central (e.g., supervisor–employee dyads, coworker conflict, team sensemaking)	Allows researchers to witness how workplace relationships are communicatively constituted in real time; reveals dominance, accommodation, alignment, or contradiction; shows co-constructed narratives	Power dynamics may silence participants; socially desirable responding; sensitive disclosures may be withheld; ethical concerns in hierarchical relationships	Lannutti (2013); Polak and Green (2016)
**4. Both Individual and Joint Interviews**	Studies of relational processes (e.g., conflict escalation, trust repair, disclosure, mediation); when comparing private and public accounts is analytically important	Enables comparison of overlap, contradiction, and discursive shifts; reveals how narratives change across contexts; captures both private sensemaking and relational performance; strong for studying privacy management and incivility dynamics	Earlier interviews may shape later ones; confidentiality management is complex; emotionally intense sessions possible; analytically demanding	Stamp (1994); Manning and Kunkel (2014b)
**5. Mixed and/or Multiple Interviews (Multiadic Designs)**	Complex organizational systems involving multiple relational constellations (e.g., supervisor–employee vs. peer–peer; leadership teams; inclusion/exclusion dynamics)	Captures how discourses shift across relational configurations; allows tracing of power, identity, and organizational narratives across units; useful for studying culture, politics, and systemic stressors	Time-consuming; participant fatigue; requires careful sequencing and design; analytic complexity increases exponentially; difficult in large organizations	Manning and Kunkel (2015); Morris (2001)

**Table 2:** Qualitative Workplace Relationship Reporting Framework (QRRF-4).

Domain	Reporting Considerations	Guiding Questions for Authors
**1. Researcher Positioning & Relational Access**	Researcher background, positionality, and access to the workplace or participants	What disciplinary, theoretical, or professional positions do the researchers bring? What relationships (past or present) exist between researchers and the workplace or participants? How were issues of power, trust, and role negotiated?
	Reflexivity and relational influence	How did researchers’ identities, assumptions, or institutional roles shape data generation, interaction, or interpretation—particularly given ongoing or sensitive workplace relationships?
**2. Relationship-Centered Study Design**	Conceptualization of workplace relationships	How are “relationships” defined and bounded (e.g., dyadic, multiadic, networked, temporally evolving)? How do relationships extend beyond formal organizational roles?
	Sampling and relational inclusion	How were participants selected in relation to one another (e.g., coworkers, supervisors/subordinates, teams)? Whose relational perspectives are included or excluded, and why?
	Contextual and organizational embedding	How is the workplace context described, including organizational culture, structure, and material conditions that shape relationships?
**3. Data Generation & Analytic Process**	Data generation methods	What qualitative methods were used (e.g., interviews, joint interviews, observations, documents, artifacts)? How did methods capture relational interaction rather than isolated accounts?
	Interactional and material sensitivity	How were communicative practices, interactional moments, or material conditions (e.g., documents, technologies, spaces) incorporated into analysis?
	Analytic approach and rigor	What analytic strategies were used (e.g., inductive, abductive, reflexive thematic analysis)? How were relational meanings traced across participants, time, or settings?
**4. Ethical & Representational Considerations**	Relational risk and confidentiality	What risks exist for participants due to relational proximity, power asymmetries, or ongoing workplace ties? How were confidentiality and anonymity managed relationally, not just individually?
	Representation and voice	How were competing or divergent relational perspectives represented? How were silences, tensions, or absences treated analytically and ethically?
	Transparency and reporting	How have ethical decisions, limitations, and trade-offs been acknowledged without displacing substantive analysis?

**Note.** The QRRF-4 is intended as a reflective reporting aid for qualitative relationship research broadly, not a prescriptive standard or measure of qualitative rigor. Authors may indicate that the checklist was consulted and addressed (e.g., in supplemental materials) as a way of signaling ethical and methodological consideration while preserving space for theory development and analysis.

## Data Availability

No new data were created or analyzed in this study.
